# New Bivariate Pareto Type II Models

**DOI:** 10.3390/e21050473

**Published:** 2019-05-06

**Authors:** Lamya Baharith, Hind Alzahrani

**Affiliations:** Department of Statistics, Faculty of Science, King Abdulaziz University, Jeddah 21551, Saudi Arabia

**Keywords:** Pareto type II, Lomax, Gaussian copula, maximum likelihood method

## Abstract

Pareto type II distribution has been studied from many statisticians due to its important role in reliability modelling and lifetime testing. In this article, we introduce two bivariate Pareto Type II distributions; one is derived from copula and the other is based on mixture and copula. Parameter Estimates of the proposed distribution are obtained using the maximum likelihood method. The performance of the proposed bivariate distributions is examined using a simulation study. Finally, we analyze one data set under the proposed distributions to illustrate their flexibility for real-life applications.

## 1. Introduction

The Pareto Type-II distribution or Pearson Type-VI distribution is called Lomax distribution introduced and studied by [1]. This distribution is commonly used in reliability and many lifetime testing studies. It is also used to analyze business data. Let T be a random variable from the Pareto type II (PII) distribution with scale parameter β and shape parameter α, then the probability density function (PDF) and the cumulative density function (CDF) of PII distribution are given respectively by

(1) $$f\left(T\right)=\frac{\alpha \beta }{{\left(1+\beta t\right)}^{\alpha +1}}\;,\;t>0$$

(2) $$F\left(T\right)=1-{\left(1+\beta t\right)}^{-\alpha },\;t>0$$

The survivor function (SF) is given by:

(3) $$S\left(T\right)={\left(1+\beta t\right)}^{-\alpha },\;t>0$$

The hazard rate function (HRF) and the cumulative hazard rate function (CHRF) are

(4) $$h\left(T\right)=\frac{\alpha \beta }{1+\beta t},\;t>0$$

(5) H(T)=αln(1+βt) , t>0

Dubey [2] showed that Pareto Type II distribution can be derived as a special case of a compound gamma distribution. Bryson [3] discussed that Lomax distribution provides an excellent alternative to classical distributions such as the exponential and Weibull distributions. Ahsanullah [4] studied the record statistics of the Lomax distribution using distributional characteristics. Balakrishnan and Ahsanullah [5] acquired some repeated relations between the moments of record values for the Lomax distribution. The Lomax distribution was used as a mixing distribution for the Poisson parameter to derive the discrete Poisson-Lomax distribution [6]. Petropoulos and Kourouklis [7] considered the estimation of a quintile of the classical marginal distribution of multivariate Lomax distribution in which the location and scale parameters are unknown. ABD [8] obtained an estimation of the Lomax parameters using maximum likelihood and Bayesian methods. Moghadam et al. [9] studied the problem of estimating the parameters of Lomax distribution based on generalization order statistics. Many scientists studied the Lomax distribution as lifetime models to provide estimates for the unknown parameters using different methods such as [10,11,12,13,14,15,16,17]. Tadikamalla [18] linked the Burr family with Lomax distribution. There are many applications for Pareto II distribution in modeling and analyzing the lifetime data in medical, engineering, and biological sciences. Examples of these applications include the mass to energy ratios in nuclear physics, Mendelian inheritance ratios in genetics, target to control precipitation in meteorology, and the stress-strength model in the context of reliability which is widely searched, see [19,20]. 

Many studies were conducted to obtain a useable multivariate or bivariate distribution for modelling real life applications. There are a number of methods in the literature that have been used successfully in constructing new multivariate distributions [21,22]. Among these, the copula method has been recognized as one of the most popular methods to construct new multivariate or bivariate distributions due to its simplicity. In addition, the dependence property of the copula method between random variables gives researchers a general structure to model multivariate distributions [23,24]. Several studies have lately introduced bivariate distributions using copula and some of these have derived by combining the mixture and copula methods [25,26,27,28,29,30,31,32].

In this article, we aim to propose new bivariate Pareto type II (BPII) distributions using copula due to the usefulness of the Pareto II distribution in many life applications and the simplicity of the copula method. The article is outlined as follows: BPII distribution derived from Gaussian copula and BPII distribution derived from mixture and Gaussian copula are proposed in Section 2. Section 3 illustrates parameter estimates of the proposed distributions. A simulation study is performed to show the flexibility of the new bivariate distributions in Section 4. Section 5 presents an analysis of one real data set to show the usefulness of the bivariate Pareto Type II distributions. The article is concluded in Section 6. 

## 2. Bivariate Pareto Type II Distributions

This section illustrates the construction of Bivariate Pareto Type II distribution derived from Gaussian copula (BPIIG) and derived from the mixture and Gaussian copula (BPIImG).

### 2.1. BPIIG Distribution

The construction of BPIIG distribution is derived using the inversion method for the PII distribution using Sklar’s theorem [23]. Therefore, the joint CDF is given by

$$F\left(T_{1}\;,T_{2}\right)=C\left[F\left(T_{1}\right),F\left(T_{2}\right)\right]$$

where $T_{1},{\;T}_{2}$ are random variables with PII distribution, and C is the Gaussian copula function with uniform margins and Pearson correlation parameter ρ∈(−1, 1) is given by

$$C={\;\Phi }_{\rho }\left(\Phi^{-1}\left(v_{1}\right),{\;\Phi }^{-1}\left(v_{2}\right),\rho \;\right)$$

$\Phi_{\rho }$ denotes the bivariate standard normal distribution function, $\Phi^{-1}$ is the inverse of univariate standard normal distribution function and $v_{1}=F\left(t_{1}\right),{\;v}_{2}=F\left(t_{2}\right)\;$, are the marginal distribution for the random variables $T_{1}{\;\mathrm{and}\;T}_{2}$, respectively.

Then, the joint PDF of $T_{1}$ and $T_{2}$ is given by

$$f\left(T_{1}\;,T_{2}\right)=C^{\prime }\left[F\left(T_{1}\right),F\left(T_{2}\right)\right]f\left(T_{1}\right)f\left(T_{2}\right)$$

where for j=1,2, $f\left(T_{j}\right)\;\mathrm{and}\;F\left(T_{j}\right)$ are given by (1) and (2), respectively, and $C^{\prime }$ is the density of the bivariate Gaussian copula obtained by differentiating C, such that

(6) $$C^{\prime }=\frac{\exp\left\{\frac{-1}{2\left(1-\rho^{2}\right)}\left(y_{1}^{2}-2{\rho y}_{1}y_{2}+y_{2}^{2}\right)\right\}}{2\pi \sqrt{1-\rho^{2}}}$$

where ${\;y}_{1}=\Phi^{-1}\left(v_{1}\right)$ and ${\;y}_{2}=\Phi^{-1}\left(v_{2}\right)$. For details see, [33,34,35].

Therefore, the joint PDF of BPII distribution with PII marginal can be rewritten as

(7) $$f\left(T_{1},T_{2}\right)=\left(\frac{\alpha_{1}\beta_{1}}{{\left(1+\beta_{1}t_{1}\right)}^{\alpha_{1}+1}}\right)\left(\frac{\alpha_{2}\beta_{2}}{{\left(1+\beta_{2}t_{2}\right)}^{\alpha_{2}+1}}\right)C^{\prime }\left(v_{1},v_{2}\right)$$

where $v_{j}=F\left(T_{j}\right),\;j=1,\;2,$ given by (1), $C^{\prime }\left(v_{1},v_{2}\right)$ given by (6). For more details, see [36,37].

**Figure 1:**
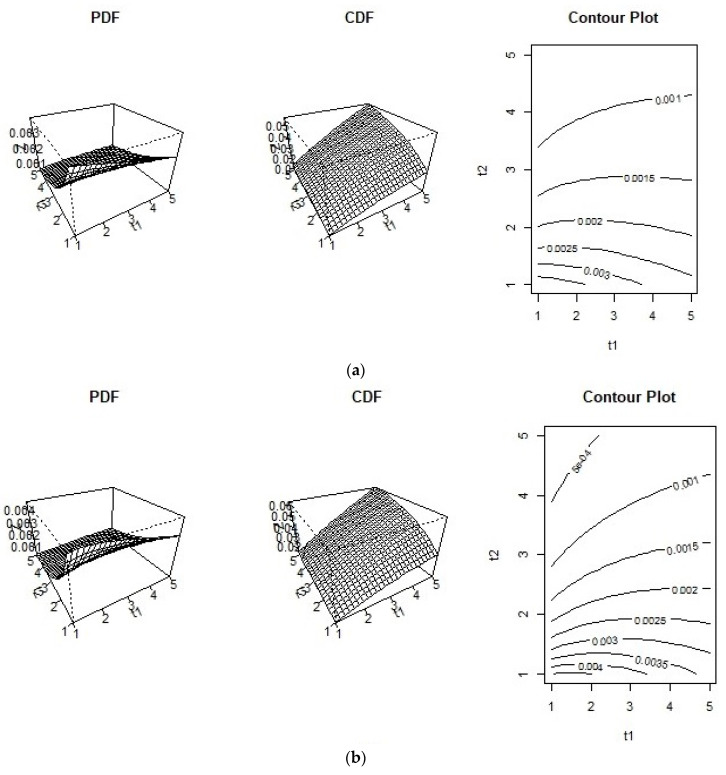
Probability density function (PDF), cumulative density function (CDF) and contour plots of the bivariate Pareto Type II models for (**a**) $\alpha_{1}=1.5\;,{\;\alpha }_{2}=2,{\;\beta }_{1}=0.01,{\;\beta }_{2}=0.03,\;\rho =0.70$, (**b**) $\alpha_{1}=1.5\;,{\;\alpha }_{2}=2,{\;\beta }_{1}=0.01,{\;\beta }_{2}=0.03,\;\rho =0.80$.

Plots of the BPIIG distribution PDF, CDF, and contour for $\alpha_{1}=1.5\;,{\;\alpha }_{2}=2,{\;\beta }_{1}=0.01,{\;\beta }_{2}=\;0.03,$ and two values of the copula parameter ρ are presented in Figure 1.

### 2.2. BPIImG Distribution

The construction of BPIImG distribution depends on the mixture representation described in [25,38,39]. The idea of mixture representation is to write the density of a random variable T on (0,∞) in the form of compound distribution as follows:

$$f\left(t\right)=\int_{\Omega }f\left(t|u\right)\;f\left(u\right)\;\mathrm{du},\;u\in \Omega ,$$

where Ω is a subset of R, U is a non-negative latent random variable following a gamma distribution with shape parameter 2 and scale parameter 1, denoted by gamma (2,1). And $f_{T|U}\left(t|u\right)$ can be written as follows

$$f\left(t|u\right)=\;\frac{h\left(t\right)}{u},u>H\left(t\right),$$

where h(t) is the HRF, and H(t) is CHRF.

That is, the mixture and copula methods are combined to obtain bivariate distribution. This is conducted by constructing a bivariate gamma distribution of latent variable $\underline{U}=\left(U_{1},U_{2}\right)$ with two marginal gamma (2,1) distributions using Gaussian copula. At first stage, we obtain a bivariate gamma distribution with only unknown correlation parameter ρ such as

(8) $$f\left(u_{1},u_{2}\right)=f\left(u_{1}\right)\;f\left(u_{2}\right)C^{\prime }\left(v_{1},v_{2}\right)$$

where $C^{\prime }\left(v_{1},v_{2}\right)$ is given by (6), $f\left(u_{j}\right)$ is the PDF of gamma (2,1), $v_{j}=F\left(u_{j}\right)$ is the CDF of gamma (2,1) given by

(9) $$F\left(u_{j}\right)=\int_{0}^{u_{j}}u_{j}e^{-u_{j}}{\;\mathrm{du}}_{j}$$

Then as a second stage, a bivariate gamma distribution in (8) is used as a mixing distribution of $T_{1},T_{2}$, assuming that $T_{1},T_{2}$ are conditionally independent given $\underline{U}$. The conditional PDF can be written as

(10) $$f\left(t_{j}{|u}_{j}\right)=\frac{\alpha_{j}\beta_{j}}{\left(1+\beta_{j}t_{j}\right)}e^{-u_{j}},{\;u}_{j}>\alpha_{j}\left(\ln\left(1+\beta_{j}t_{j}\right)\right)$$

And then integrate over the latent variables $\underline{U}$ to obtain the joint PDF of BPIImG distribution is as follows

(11) $$f\left(t_{1},t_{2}\right)=\int_{H\left(t_{2}\right)}^{\infty }\int_{H\left(t_{1}\right)}^{\infty }\overset{2}{\underset{j=1}{\prod }}\left[\frac{\alpha_{j}\beta_{j}}{\left(1+\beta_{j}t_{j}\right)}{\;e}^{-u_{j}}\right]C^{\prime }\left(v_{1},v_{2}\right){\;\mathrm{du}}_{1}{\mathrm{du}}_{2},$$

using the above two stages method will help in the model analysis, because we can estimate the correlation parameter ρ from the first stage (i.e., the bivariate gamma distribution). Then, estimate the other parameters from the second stage (i.e., the conditional density functions $f\left(t_{j}{|u}_{j}\right)$).

## 3. Estimation

### 3.1. Estimation for BPIIG Parameters

If $T_{i}=\left(T_{1i},T_{2i}\right)$, is a bivariate random sample from BPII distribution with probability function in (7), then the likelihood function is

$$L\left(\theta |T_{1},T_{2}\right)=\overset{n}{\underset{i=1}{\prod }}f\left(t_{1i},t_{2i}\right)=\overset{2}{\underset{j=1}{\prod }}\overset{n}{\underset{i=1}{\prod }}\left[\left(\frac{\alpha_{j}\beta_{j}}{{\left(1+\beta_{j}t_{\mathrm{ji}}\right)}^{\alpha_{j}+1}}\right)\;\right]C^{\prime }\left(v_{1},v_{2}\right)$$

where $\theta =(\beta_{1},\alpha_{1},\beta_{2},\alpha_{2},\;\rho )$. The log-likelihood function is given by

(12) $$\ell =\overset{2}{\underset{j=1}{\sum }}n\ln\alpha_{j}+n\;\ln\beta_{j}-\left(\alpha_{j}+1\right)\sum_{i=1}^{n}\ln\left(1+\beta_{j}t_{\mathrm{ji}}\right)+\sum_{i=1}^{n}\left[\ln\left(C^{\prime }\left(v_{1},v_{2}\right)\right)\right]$$

The maximum likelihood (ML) estimates are obtained by differentiating (12) with respect to $\beta_{1},\alpha_{1},\beta_{2},\alpha_{2},\;\mathrm{and}\;\rho$. Then, the first partial derivatives are as follows:

(13) $$\begin{array}{c} \frac{\partial \ell }{\partial \alpha_{j}}=\frac{n}{\alpha_{j}}-\sum_{i=1}^{n}\ln\left(1+\beta_{j}t_{\mathrm{ji}}\right)=0,\\ \frac{\partial \ell }{\partial \beta_{j}}=\frac{n}{\beta_{j}}-\left(\alpha_{j}+1\right)\overset{n}{\underset{i=1}{\sum }}\frac{t_{\mathrm{ji}}}{\left(1+\beta_{j}t_{\mathrm{ji}}\right)}=0\\ \frac{\partial \ell }{\partial \rho }=0\underset{\Rightarrow }{}\hat{\rho }=\overset{n}{\underset{i=1}{\sum }}\frac{y_{1i}{\;y}_{2i}}{n} \end{array}\}$$

The ML estimates of $\beta_{1},\alpha_{1},\beta_{2},\alpha_{2},\;\mathrm{and}\;\rho$ can be obtained by solving (13) numerically.

In addition, we can obtain approximate confidence interval (CI) of the parameters $\beta_{1},\alpha_{1},\beta_{2},\alpha_{2},\;\mathrm{and}\;\rho$ by using large sample theory and ML estimates of asymptotic distribution. That is, $\theta =(\beta_{1},\alpha_{1},\beta_{2},\alpha_{2},\;\rho )\sim \mathrm{multivariate}\;\mathrm{normal}\left(\theta ,I^{-1}\left(\theta \right)\right)$, where $I^{-1}$ is the inverse of the observed information matrix given by

$$I^{-1}\left(\theta \right)={\left(\begin{array}{ccccc} \frac{\partial^{2}\ell }{\partial \alpha_{1}^{2}} & \frac{\partial^{2}\ell }{\partial \alpha_{1}\partial \alpha_{2}} & \frac{\partial^{2}\ell }{\partial \alpha_{1}\partial \beta_{1}} & \frac{\partial^{2}\ell }{\partial \alpha_{1}\partial \beta_{2}} & \frac{\partial^{2}\ell }{\partial \alpha_{1}\partial \rho }\\ \frac{\partial^{2}\ell }{\partial \alpha_{2}\partial \alpha_{1}} & \frac{\partial^{2}\ell }{\partial \alpha_{2}^{2}} & \frac{\partial^{2}\ell }{\partial \alpha_{2}\partial \beta_{1}} & \frac{\partial^{2}\ell }{\partial \alpha_{2}\partial \beta_{2}} & \frac{\partial^{2}\ell }{\partial \alpha_{2}\partial \rho }\\ \frac{\partial^{2}\ell }{\partial \beta_{1}\partial \alpha_{1}} & \frac{\partial^{2}\ell }{\partial \beta_{1}\partial \alpha_{2}} & \frac{\partial^{2}\ell }{\partial \beta_{1}^{2}} & \frac{\partial^{2}\ell }{\partial \beta_{1}\partial \beta_{2}} & \frac{\partial^{2}\ell }{\partial \beta_{1}\partial \rho }\\ \frac{\partial^{2}\ell }{\partial \beta_{2}\partial \alpha_{1}} & \frac{\partial^{2}\ell }{\partial \beta_{2}\partial \alpha_{2}} & \frac{\partial^{2}\ell }{\partial \beta_{2}\partial \beta_{1}} & \frac{\partial^{2}\ell }{\partial \beta_{2}^{2}} & \frac{\partial^{2}\ell }{\partial \beta_{2}\partial \rho }\\ \frac{\partial^{2}\ell }{\partial \rho \partial \alpha_{1}} & \frac{\partial^{2}\ell }{\partial \rho \partial \alpha_{2}} & \frac{\partial^{2}\ell }{\partial \rho \partial \beta_{1}} & \frac{\partial^{2}\ell }{\partial \rho \partial \beta_{2}} & \frac{\partial^{2}\ell }{\partial p^{2}} \end{array}\right)}^{-1}$$

The second derivative of (13) with respect to the parameters are provided in the Appendix A. Therefore, 100(1−γ)% approximate CI for the parameters ${\;\beta }_{1},\alpha_{1},\beta_{2},\alpha_{2},\;\mathrm{and}\;\rho$ for j=1,2 are given by

$${\hat{\alpha }}_{j}\mp z_{\gamma /2}\sqrt{\mathrm{var}\left({\hat{\alpha }}_{j}\right)}$$

$${\hat{\beta }}_{j}\mp z_{\gamma /2}\sqrt{\mathrm{var}\left({\hat{\beta }}_{j}\right)}$$

$$\hat{\rho }\mp z_{\gamma /2}\sqrt{\mathrm{var}\left(\hat{\rho }\right)}$$

where: $z_{\gamma /2}$ is the upper (γ/2)% of the standard normal distribution. The CI of the parameters could be adjusted for the lower bound using the method in [40].

### 3.2. Estimation for BPIImG Parameters

If $T_{i}=\left(T_{1i},T_{2i}\right)$ is a bivariate random sample of size n from BPII distribution, and $\underline{U}=\left(U_{1i},U_{2i}\right),\;i=1,\ldots ,\;n$ is a random sample from bivariate gamma distribution, then the log-likelihood function can be written as

(14) $$\ell \left(\theta |T_{1},T_{2},U_{1},U_{2}\right)=\overset{2}{\underset{j=1}{\sum }}n\ln\alpha_{j}+n\;\ln\beta_{j}-\overset{n}{\underset{i=1}{\sum }}u_{\mathrm{ji}}-\overset{n}{\underset{i=1}{\sum }}\ln\left(1+\beta_{j}t_{\mathrm{ji}}\right)+\sum_{i=1}^{n}\left[\ln\left(C^{\prime }\left(v_{1},v_{2}\right)\right)\right]$$

where $u_{j}>\alpha_{j}\left(\ln\left(1+\beta_{j}t_{j}\right)\right)$, and $v_{j}=F\left(u_{j}\right)$ given by (9).

The ML estimates of $\theta =\left(\beta_{1},\alpha_{1},\beta_{2},\alpha_{2},\rho \right)$ can be obtained by differentiating (14) with respect to $\beta_{1},\alpha_{1},\beta_{2},\alpha_{2},\;\mathrm{and}\;\rho$ and solving the following equations:

(15) $$\begin{array}{c} \frac{\partial \ell }{\partial \alpha_{j}}=\frac{n}{\alpha_{j}}-\frac{\partial \sum_{i=1}^{n}u_{\mathrm{ji}}}{\partial \alpha_{j}}=0,\\ \frac{\partial \ell }{\partial \beta_{j}}=\frac{n}{\beta_{j}}-\overset{n}{\underset{i=1}{\sum }}\frac{t_{\mathrm{ji}}}{\left(1+\beta_{j}t_{\mathrm{ji}}\right)}-\frac{\partial \sum_{i=1}^{n}u_{\mathrm{ji}}}{\partial \beta_{j}}=0\\ \frac{\partial \ell }{\partial \rho }=0\overset{}{\Rightarrow }\hat{\rho }=\overset{n}{\underset{i=1}{\sum }}\frac{y_{1i}{\;y}_{2i}}{n} \end{array}\}$$

The nonlinear system of equations in (15) can be solved numerically to obtain the ML estimates of $\beta_{1},\alpha_{1},\beta_{2},\alpha_{2},\;\mathrm{and}\;\rho$.

## 4. Simulation Study

Monte Carlo simulation studies were conducted to estimates the parameters for BPIIG and BPIImG distributions. In addition, we investigated and compared the performance of the ML estimates at different sample sizes; n = (80, 150, 300, 350, 400) with the selected values of the parameters, $\left(\beta_{1}=2.1,\alpha_{1}=1.1,\beta_{2}=2.5,\alpha_{2}=1.5\right),\;$ keeping the copula parameter ρ=(0.3, 0.70, 0.80). 

### 4.1. ML Estimates of BPIIG

ML parameter estimates of the BPIIG distribution are shown in Table 1 along with the corresponding relative mean square error (RMSE).

The results in Table 1 show that as the sample size increases, the RMSE of the parameters estimates become smaller. In addition, most parameters have better estimates and smaller RMSEs when the copula parameter equal to 0.80.

### 4.2. ML Estimates of BPIImG 

Parameter estimates of BPIImG distribution using ML methods are illustrated in Table 2. In addition, the average estimates along with their RMSE over 1000 replication are reported. 

The results reported in Table 2 indicate that the RMSE of the parameter estimates decreases as the sample size increases. Also, we obtained better estimates of the parameters with smaller RMSE especially the estimate of ρ when the copula parameter is equal to 0.80 and the sample size is more than 150.

### 4.3. Models Comparison

We compared the flexibility of the BPIIG and BPIImG distributions based on RMSE, Akaike information criterion (AIC), and Bayesian information criterion (BIC) values. The results in Table 3 indicate that the BPIImG distribution has lower values of AIC and BIC. Therefore, we conclude that BPIImG distribution is more flexible and perform better than BPIIG.

## 5. Data Analysis

The American football league data obtained from the matches played on three consecutive weekends in 1986 have two variables $T_{1}$ and $T_{2}$ where; $T_{1}$ is the game time the first fields scored when the ball kicks between goalposts and $T_{2}$ is the game time the first touchdown is scored, see [41]. The histogram and the scatter plots of $T_{1}$ and $T_{2}$ are right skewed and positively correlated [29]. The sample Spearman correlation coefficient between $T_{1}$ and $T_{2}$ is 0.804 which allows using the proposed BPII distribution to model this bivariate data. Also, we conducted goodness of fit test by fitting the marginals only, see [42].

**Figure 2:**
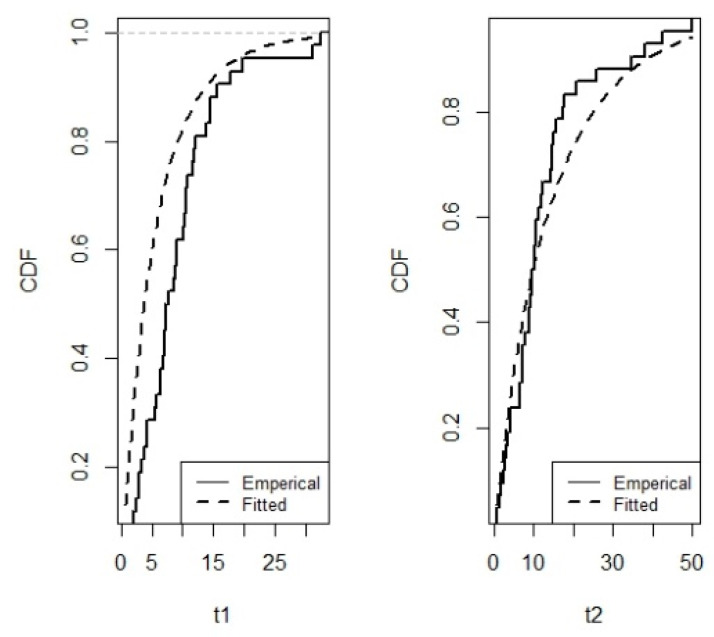
The plots of the fitted and the empirical CDF for the two marginals based on maximum likelihood (ML) estimate respectively.

That is, the PII distribution is fitted to the marginals and the ML estimates of the parameters are: ${\hat{\beta }}_{1}=0.011,{\hat{\alpha }}_{1}=9.519,{\hat{\beta }}_{2}=0.0141,{\hat{\alpha }}_{2}=5.3778$. The plots of the fitted and the empirical CDF for the two marginals based on ML estimates are illustrated in Figure 2. The Kolmogorov-Smirnov (K-S) test values and the associated *p*-values (reported in brackets) for $T_{1}$ and $T_{2}$ are 0.1521(0.2855) and 0.1355(0.3884). 

Hence, the K-S test along with the plots of the fitted and the empirical CDF in Figure 2 indicate that the BPII distribution has an appropriate fit for this bivariate data. In addition, the Gaussian copula is appropriate for this data as indicated in [29]. For more details, see [43].

Table 4 reports the ML estimates of the parameters along with the standard error (SE) of the BPIIG and BPIImG parameters. It can be seen from Table 4 that the AIC of BPIImG distribution is smaller compared to BPIIG distribution. This indicates that BPIImG distribution is more appropriate for this data.

The model’s comparison illustrated in [29] is re-conducted to compare BPIIG and BPIImG with Bivariate expatiated Pareto derived from the mixture and Gaussian copula (BEPmG), bivariate exponentiated generalized Weibull-Gompertz distribution (BEGWG) studied by [44], and bivariate exponentiated Gompertez distribution (BEG) using the same real data set.

The results in Table 5 show that BPIImG distribution has the lowest AIC, and BIC values compared the BEPmG, BEGWG, BEG and BPIIG distributions. Therefore, BPIImG provides a more appropriate and flexible fit for this data set.

## 6. Conclusions

In this article, we introduced BPIIG and BPIImG distributions. Parameter estimates of the proposed bivariate distributions are obtained using the ML method. A simulation study is carried out to show the performance of the proposed bivariate distributions. We concluded that the BPIImG distribution is more flexible and performs better than the BPIIG distribution. A real lifetime data is analyzed, and the results showed that the BPIImG distribution provides a more suitable fit than the BPIIG, BEPmG, BEG, and BEGWG distributions.

## Figures and Tables

**Table 1:** Maximum likelihood (ML) average estimates for the parameters of bivariate Pareto Type II distribution based on Gaussian copula (BPIIG) and the corresponding relative mean square error (RMSE).

Sample Size	Parameters	ML ρ=0.30		ML ρ=0.70		ML ρ=0.80	
		Mean	RMSE	Mean	RMSE	Mean	RMSE
80	${\hat{\alpha }}_{1}$	1.2137	0.1321	1.2076	0.1114	1.1923	0.0881
	${\hat{\alpha }}_{2}$	1.7906	0.4042	1.6979	0.2457	1.6937	0.4678
	${\hat{\beta }}_{1}$	2.1038	0.3529	2.0866	0.2779	2.1132	0.3038
	${\hat{\beta }}_{2}$	2.4345	0.4989	2.4847	0.3783	2.5225	0.3113
	$\hat{\rho }$	0.2945	0.0369	0.6900	0.0161	0.7995	0.0019
150	${\hat{\alpha }}_{1}$	1.1626	0.0508	1.1519	0.0451	1.15828	0.0450
	${\hat{\alpha }}_{2}$	1.6207	0.1151	1.6233	0.1085	1.5980	0.0952
	${\hat{\beta }}_{1}$	2.0828	0.1743	2.0986	0.1627	2.0909	0.1625
	${\hat{\beta }}_{2}$	2.4643	0.2409	2.4629	0.2282	2.5114	0.2267
	$\hat{\rho }$	0.3001	0.0187	0.6975	0.0022	0.8002	0.0009
300	${\hat{\alpha }}_{1}$	1.1238	0.0172	1.1281	0.0159	1.1155	0.0162
	${\hat{\alpha }}_{2}$	1.5469	0.0406	1.5494	0.0364	1.5320	0.0316
	${\hat{\beta }}_{1}$	2.0901	0.0825	2.1004	0.0749	2.1075	0.0777
	${\hat{\beta }}_{2}$	2.4974	0.1193	2.5087	0.1134	2.4988	0.1020
	$\hat{\rho }$	0.2983	0.0091	0.6987	0.0010	0.8001	0.0004
350	${\hat{\alpha }}_{1}$	1.1268	0.0178	1.1244	0.0147	1.1145	0.0129
	${\hat{\alpha }}_{2}$	1.5393	0.0315	1.5396	0.0309	1.5304	0.0265
	${\hat{\beta }}_{1}$	2.0989	0.0760	2.1006	0.0670	2.1023	0.0610
	${\hat{\beta }}_{2}$	2.5078	0.1044	2.5116	0.0973	2.4911	0.0852
	$\hat{\rho }$	0.2985	0.0073	0.6988	0.0009	0.7998	0.0003
400	${\hat{\alpha }}_{1}$	1.1134	0.0131	1.1222	0.0128	1.1135	0.0111
	${\hat{\alpha }}_{2}$	1.5338	0.0264	1.5346	0.0260	1.5241	0.0221
	${\hat{\beta }}_{1}$	2.1221	0.0673	2.0982	0.0581	2.1012	0.0532
	${\hat{\beta }}_{2}$	2.4999	0.0867	2.5073	0.0846	2.4989	0.0721
	$\hat{\rho }$	0.3009	0.0066	0.6988	0.0008	0.7995	0.0003

**Table 2:** ML average estimates for the parameters of bivariate Pareto Type II distribution based on mixture and Gaussian copula (BPIImG) and the corresponding RMSE.

Sample Size	Parameters	ML ρ=0.30		ML ρ=0.70		ML ρ=0.80	
		Mean	RMSE	Mean	RMSE	Mean	RMSE
80	${\hat{\alpha }}_{1}$	1.2204	0.1456	1.2036	0.0532	1.1812	0.1163
	${\hat{\alpha }}_{2}$	1.8343	0.7488	1.6036	0.0419	1.6125	0.2962
	${\hat{\beta }}_{1}$	2.0930	0.3395	2.1039	0.1772	2.1818	0.3749
	${\hat{\beta }}_{2}$	2.4204	0.2852	2.5019	0.1168	2.5054	0.3149
	$\hat{\rho }$	0.3018	0.0326	0.7024	0.0043	0.7987	0.0019
150	${\hat{\alpha }}_{1}$	1.1460	0.0440	1.1551	0.0461	1.1523	0.0476
	${\hat{\alpha }}_{2}$	1.5988	0.1096	1.5901	0.0412	1.5909	0.2826
	${\hat{\beta }}_{1}$	2.1260	0.1941	2.1152	0.0923	2.1291	0.1871
	${\hat{\beta }}_{2}$	2.5215	0.1630	2.4963	0.1161	2.5292	0.1572
	$\hat{\rho }$	0.2998	0.0184	0.7022	0.0025	0.7998	0.0010
300	${\hat{\alpha }}_{1}$	1.1329	0.0213	1.1319	0.0214	1.1235	0.0193
	${\hat{\alpha }}_{2}$	1.5480	0.0484	1.5620	0.0478	1.5400	0.0384
	${\hat{\beta }}_{1}$	2.0869	0.0926	2.1035	0.0914	2.1037	0.0891
	${\hat{\beta }}_{2}$	2.4916	0.0778	2.4976	0.0767	2.5059	0.0748
	$\hat{\rho }$	0.3018	0.0089	0.6997	0.0013	0.7992	0.0005
350	${\hat{\alpha }}_{1}$	1.1217	0.0169	1.1158	0.0154	1.1145	0.0151
	${\hat{\alpha }}_{2}$	1.5374	0.0335	1.5263	0.0307	1.5368	0.0295
	${\hat{\beta }}_{1}$	2.1065	0.0844	2.1318	0.0795	2.1181	0.0743
	${\hat{\beta }}_{2}$	2.5030	0.0701	2.5450	0.0668	2.5022	0.0624
	$\hat{\rho }$	0.3024	0.0077	0.7003	0.0011	0.7984	0.0005
400	${\hat{\alpha }}_{1}$	1.1247	0.0150	1.1156	0.0130	1.1177	0.0136
	${\hat{\alpha }}_{2}$	1.5421	0.0287	1.5416	0.0332	1.5345	0.0286
	${\hat{\beta }}_{1}$	2.0948	0.0699	2.1012	0.0676	2.1013	0.0634
	${\hat{\beta }}_{2}$	2.4936	0.0587	2.4825	0.0568	2.5035	0.0533
	$\hat{\rho }$	0.2983	0.0064	0.6993	0.0009	0.7998	0.0004

**Table 3:** RMSE, Akaike information criterion (AIC), and Bayesian information criterion (BIC) for BPIIG and BPIImG distributions with ρ=0.80.

Model	n	AIC	BIC
BPIIG	300	1054.0	1072.5
	400	1402.6	1422.6
BPIImG	300	672.0	690.5
	400	876.6	896.6

**Table 4:** ML estimates, standard error and AIC for BPIIG and BPIImG distributions.

Model	Par.	ML Estimate	SE	AIC
BPIIG	${\hat{\beta }}_{1}$	0.0117	0.01	526.8
	${\hat{\alpha }}_{1}$	9.9106	5.15	
	${\hat{\beta }}_{2}$	0.0457	0.02	
	${\hat{\alpha }}_{2}$	2.2948	0.95	
	$\hat{\rho }$	0.9236	0.02	
BPIImG	${\hat{\beta }}_{1}$	0.0122	0.01	264.1
	${\hat{\alpha }}_{1}$	9.5197	5.68	
	${\hat{\beta }}_{2}$	0.0159	0.01	
	${\hat{\alpha }}_{2}$	4.7856	2.97	
	$\hat{\rho }$	0.8781	0.03	

**Table 5:** Reports the ML estimates, the maximized log likelihood values (ℓ), Akaike information criterion (AIC) for the bivariate exponentiated Gompertez (BEG), bivariate exponentiated generalized Weibull-Gompertz (BEGWG), Bivariate expatiated Pareto derived from the mixture and Gaussian copula (BEPmG) and BPIIG and BPIImG distributions.

Models	ML Estimates					ℓ	AIC	BIC
BEG	${\hat{\alpha }}_{1}$ = 0.04	${\hat{\alpha }}_{2}$ = 0.53	${\hat{\alpha }}_{3}$ = 1.04	$\hat{\lambda }=0.79$		370.41	748.82	755.77
BEGWG	${\hat{\alpha }}_{1}=0.04$	${\hat{\alpha }}_{2}$ = 0.19	${\hat{\alpha }}_{3}$ = 0.41			354.03	714.06	719.80
BEPmG	${\hat{\theta }}_{1}=9.95$	${\hat{\lambda }}_{1}=1.38$	${\hat{\theta }}_{2}=8.01$	${\hat{\lambda }}_{2}=1.14$	$\hat{\rho }$ = 0.927	252.27	514.56	523.25
BPIIG	${\hat{\alpha }}_{1}=9.91$	${\hat{\beta }}_{1}=0.01$	${\hat{\alpha }}_{2}=2.30$	${\hat{\beta }}_{2}=0.05$	$\hat{\rho }$ = 0.924	286.71	526.83	535.52
BPIImG	${\hat{\alpha }}_{1}=9.52$	${\hat{\beta }}_{1}=0.01$	${\hat{\alpha }}_{2}=4.79$	${\hat{\beta }}_{2}=0.02$	$\hat{\rho }$ = 0.878	218.32	446.63	455.32

## Appendix A

The second partial derivatives will be simplified as follows:

$$I_{11}=I_{22}=-E\left[\frac{\partial^{2}\ell }{\partial \alpha_{j}^{2}}\right]=\frac{n}{\alpha_{j}^{2}}\;I_{12}=I_{21}=-E\left[\frac{\partial^{2}\ell }{\partial \alpha_{1}\partial \alpha_{2}}\right]=0$$

$$I_{13}=I_{31}=-E\left[\frac{\partial^{2}\ell }{\partial \alpha_{1}\partial \beta_{1}}\right]=-\overset{n}{\underset{i=1}{\sum }}\frac{t_{1i}}{\left(1+\beta_{1}t_{1j}\right)}\;$$

$$I_{33}=I_{44}=-E\left[\frac{\partial^{2}\ell }{\partial \beta_{j}^{2}}\right]=\frac{n}{\beta_{j}^{2}}+\left(\alpha_{j}+1\right)\overset{n}{\underset{i=1}{\sum }}\frac{t_{\mathrm{ji}}}{{\left(1+\beta_{j}t_{\mathrm{ji}}\right)}^{2}}$$

$$I_{14}=I_{41}=-E\left[\frac{\partial^{2}\ell }{\partial \alpha_{1}\partial \beta_{2}}\right]=-E\left[\frac{\partial^{2}\ell }{\partial \beta_{2}\partial \alpha_{1}}\right]=0\;I_{24}=I_{42}=-E\left[\frac{\partial^{2}\ell }{\partial \alpha_{2}\partial \beta_{1}}\right]=-E\left[\frac{\partial^{2}\ell }{\partial \beta_{1}\partial \alpha_{2}}\right]=0$$

$$I_{34}=I_{43}=-E\left[\frac{\partial^{2}\ell }{\partial \beta_{1}\partial \beta_{2}}\right]=0$$

$$I_{15}=I_{51}=I_{25}=I_{52}=-E\left[\frac{\partial^{2}\ell }{\partial \alpha_{j}\partial \rho }\right]=0\;\;I_{35}=I_{53}=I_{45}=I_{54}=-E\left[\frac{\partial^{2}\ell }{\partial \beta_{j}\partial \rho }\right]=0\;I_{55}=-E\left[\frac{\partial^{2}\ell }{\partial \rho^{2}}\right]$$
